# Lipofuscin: a key compound in ophthalmic practice

**DOI:** 10.22336/rjo.2021.23

**Published:** 2021

**Authors:** Edward Florian Călin, Stella Ioana (Patoni) Popescu, Corina Cristina (Coman) Cernat, Cristina Patoni, Marius-Nicolae Popescu, Ovidiu Mușat

**Affiliations:** * Department of Ophthalmology, “Dr. Carol Davila” Central Military Emergency University Hospital, Bucharest, Romania; ** “Victor Babeş” University of Medicine and Pharmacy, Timișoara, Romania; *** Department of Gastroenterology, “Dr. Carol Davila” Central Military Emergency University Hospital, Bucharest, Romania; **** Department of Rehabilitation, Elias Emergency University Hospital, Bucharest, Romania

**Keywords:** lipofuscin, A2E, retinal pigment epithelium, fundus autofluorescence

## Abstract

Lipofuscin is an intracellular aging pigment with fluorescent properties, found in retinal pigment epithelium cells of the eye. It is the main fluorophore used in fundus autofluorescence imaging techniques to diagnose, describe, and follow retinal disease. Lipofuscin forms by incomplete lysosomal degradation of cellular material previously subjected to oxidative changes. A2E is the most studied fluorescent component of lipofuscin, but most of its composition remains unknown. Lipofuscin is photoreactive, generating reactive oxygen species, which may explain its role in disease development. Further knowledge is needed concerning lipofuscin genesis, biochemical composition, fluorescent compounds, and role in pathogenesis of retinal degenerative disease.

## Introduction

Fundus autofluorescence is an imaging method used in ophthalmology to visualize anatomic and functional aspects of the photoreceptor/ retinal pigment epithelium (RPE) complex, an essential element of healthy vision. The technique uses fluorescent properties of the natural fluorophore lipofuscin, an aging pigment, that progressively accumulates within RPE cells. A2E has long been considered one of the main components of lipofuscin with fluorescent properties and a noteworthy role in retinal degenerative disease. Recent knowledge about A2E has shown that this compound, although important, does not completely explain the fluorescent and pathologic properties of lipofuscin [**1**-**3**] and further research is needed in order to fully describe the exact molecular components of lipofuscin and its biochemical properties.

## Materials and methods

***Lipofuscin, an aging pigment***

Lipofuscin is the name given to nondegradable aggregates consisting of lipids and proteins that progressively accumulate throughout life within the lysosomal compartment of various cells of the body [**4**]. It is a ubiquitous yellow-brown aging pigment that is stored particularly within postmitotic cells with high metabolic activity. This includes cardiac myocytes, neurons and RPE. Lipofuscin is heterogenous in origin, composition, and biochemical qualities, depending on the cell type in which it accumulates. 

We believe it is important to concisely mention ceroid, an intracellular pigment like lipofuscin. Unlike the latter, ceroid is strictly a pathological pigment and is present in a series of central nervous system degenerative disease called neuronal ceroid lipofuscinoses [**5**].

***Lipofuscin and retinal pigment epithelium***

Lipofuscin forms clusters of granules inside the RPE [**4**]. These granules are membrane-bound and lay in the basal half of RPE cells. They measure between 1 to 2 μm in diameter, with undulated or scalloped outlines [**6**]. In aged individuals, lipofuscin granules may conjugate with melanin forming aggregates called melanolipofuscin [**7**]. Lipofuscin and melanolipofuscin granules may occupy up to one third of the cytoplasmic space of RPE cells in persons aged over 70 [**8**]. 

Lipofuscin content of RPE increases with age until about 70 years and then plateaus or decreases (probably by removal of atrophic RPE cells) with a concomitant decrease in melanosomes and increase in melanolipofuscin granules [**9**]. The topographic distribution of lipofuscin across the retina appears to follow that of rod cells [**7**,**9**]. 

Lipofuscin granules inside RPE cells demonstrate a characteristic orange-red or golden-yellow autofluorescence when excited with ultraviolet or blue light [**10**]. These spectral properties allow for noninvasive imaging of lipofuscin content of the RPE.

***Lipofuscin formation***

Lipofuscin may be formed by two ways: either by autophagy (a mechanism essential for homeostasis maintenance in which a cell degrades its own intracellular organelles, such as mitochondria) or by phagocytosis of other cellular components, as in the case of the RPE, which functions to phagocytose photoreceptor external segments [**4**,**11**,**12**]. A significant amount of RPE lipofuscin may in fact be due to autophagy as in vitro studies have shown that lipofuscin-like granules are present in RPE cells even in the absence of phagocytic challenge of photoreceptor outer segment [**13**].

***Biochemical composition of lipofuscin***

The biochemical composition of lipofuscin is complex, comprising variable amounts of lipids, proteins, carbohydrates and metal ions, its composition depending on the tissue of origin. Incomplete degradation of cellular material within the lysosomal compartment is due to oxidative changes of these compounds either by normal cell metabolism or oxidative stress [**9**]. The photoreceptor/ RPE complex is considered highly susceptible to oxidative modification due to its important exposure to sunlight, elevated oxygen levels in circulating blood and high content of polyunsaturated fatty acids (PUFAs) in mitochondrial and cell membranes [**9**,**14**]. These oxidized products are further modified within lysosomes [**9**]. Evidence that these alterations take place is the fact that the spectral properties of lipofuscin fluorophores differ from those of their oxidized precursors [**15**].

Reactive oxygen species from cell metabolism or oxidative stress induce lipid peroxidation, resulting in lipid hydroperoxides or cyclic peroxides, which further degrade to form carbonyl compounds, mainly aldehydes [**15**]. These aldehydes represent the precursors of fluorescent products. Malondialdehyde (MDA) and 4-hydroxynonenal (HNE) have been most extensively studied. MDA further reacts with amino acids in low-pH conditions to form Schiff bases with aminopropane structure (-N=CH-CH=CH-NH-). These Schiff bases have emission in the 450-470 nm region when excited by ultraviolet light. This could explain some of the fluorescent properties of lipofuscin. Schiff bases are also created by interaction between reducing sugars and amino compounds, a process called nonenzymatic glycosylation or Maillard reaction. Schiff bases are then converted to ketoamines, called Amadori products, which are further degraded to unsaturated carbonyls, the analogues of lipid peroxidation products. Although the list of age-pigment like fluorophores is more extensive than our description [**15**,**16**], all appear to form through a common conjugation between amino compounds and carbonyls.

Despite the numerous attempts over the last decades to describe the complex composition of lipofuscin in RPE cells, its constituents remain mostly unknown. A proteomic study done by Ng et al. [**17**] have shown that that purified lipofuscin granules from RPE cells contain only small amounts of protein and that modified lipids from outer segment and mitochondrial membranes are the major contributors, both to lipofuscin composition and to its presumed phototoxic activity. 

***A2E – A key component of lipofuscin?***

To characterize the chromatographic and spectral properties of the fluorescent components of human RPE lipofuscin, Eldred and Katz [**18**] separated lipofuscin from donor eyes into 3 phases using methanol and chloroform: a polar (methanol-water) phase, a nonpolar (chloroform) phase and an insoluble fraction. The polar phase emitted only a faint fluorescence and was not further characterized. Using thin layer chromatography, they analyzed the nonpolar fraction and identified 10 fluorophores with different emission spectra. It is important to mention that not all the age-related fluorescent material was extracted and that the fluorescent insoluble fraction increased with age. 

Later, Eldred and Lasky [**19**] showed that the main orange-emitting fluorophore is N-retinylidene-N-retinylethanolamine, a pyridinium bis-retinoid synthetized using 2 molecules of all-trans-retinal and 1 molecule of ethanolamine (A2E). A2E has an amphiphilic structure responsible for its detergent-like action in membrane disruption. Its quaternary amine structure also inhibits lysosomal action by binding to certain lysosomal enzymes [**19**]. In addition to A2E, lipofuscin contains molecules structurally related to A2E resulting from autooxidation or photooxidation of A2E [**20**]. The composition of the insoluble part of lipofuscin remains unknown. This fraction may also play a significant role in photoreactivity of lipofuscin granules [**21**].

***Spectral characteristics of lipofuscin***

Boulton et al. [**22**] studied the spectral characteristics of lipofuscin granules and melanosomes in human RPE cells with respect to donor age. When excited at 364 nm, lipofuscin granules showed a broad emission spectrum with a peak at 600 nm. The same emission peak was observed when an excitation of 476 nm was employed, a value like the excitation employed by in vivo autofluorescence systems [**9**]. When comparing emission spectra from different age groups, three other spectral regions were of interest: a blue-green shoulder at 470 nm and a green-yellow shoulder at 550 nm were evident in 4–29-year-old and 30–49-year-old but absent in > 50-year-old. A third red shoulder at 680 nm was consistent in the > 50-year-old group. Emission in blue decreased with increasing age while emission in red increased. The fluorescence intensity of lipofuscin granules increased with increasing age. Both the emission and the excitation spectra peaks were 40% higher in older individuals when compared with younger ones. Melanolipofuscin has spectral characteristics intermediate between those of lipofuscin and melanosomes [**9**].

***Lipofuscin phototoxicity***

In conditions simulating in vivo conditions of human RPE cells (light exposure and aerobic conditions relating to high oxygen tension of circulating blood), RPE lipofuscin shows notable photoreactivity. Lipofuscin has phototoxic potential that appears to be wavelength-dependent: exposure of lipofuscin-containing cells to short-wavelength visible light (390-550 nm) caused lipid peroxidation, with increased levels of MDA and HNE, protein oxidation with formation of protein carbonyl, loss of lysosomal integrity, cytoplasmic vacuolation and membrane blebbing. The cytologic damage culminated in cell death. Exposure to wavelengths of 550 to 800 nm did not show any adverse effects [**23**]. The photooxidative damage appears to be greater in aged RPE because of a greater number of lipofuscin granules as well as an overall increased photoreactivity of lipofuscin with increasing age [**21**]. 

Lipofuscin generates reactive oxygen species (ROS), including singlet oxygen (1O2), superoxide anion (O2-) and hydrogen peroxide (H2O2), as well as lipid hydroperoxides and MDA, which are suggestive of lipid peroxidation. Photoreactivity is also wavelength-dependent [**24**,**25**]. 

Because of its photoreactivity, lipofuscin may contribute to the pathogenesis of age-related macular degeneration (AMD) [**9**], its accumulation in RPE cells concurring with the development of AMD. Furthermore, retinal areas with high amounts of lipofuscin seem the most susceptible to degeneration [**26**]. The most studied component and potential phototoxic component of lipofuscin is A2E. The evidence has shown that A2E has weak photoreactivity and its contribution to RPE cytotoxicity is arguable [**24**]. Another source of photoreactivity may be represented be A2E-derived molecules, such as the epoxide nonaoxirane [**27**]. The insoluble fractions of lipofuscin may also represent an important source of photoreactivity [**4**,**24**].

***Lipofuscin and retinal degenerative disease***

Autofluorescence imaging of the retina shows elevated levels of lipofuscin associated with retinal degeneration [**26**,**28**,**29**]. Across various retinal degenerative disease, it is still difficult to determine if lipofuscin is the causative agent of disease or its consequence. In the case of AMD, there is significant evidence of the pathogenic role of lipofuscin in this disease, either by exhibiting oxidative stress and chronic dysfunction of RPE cells [**4**,**24**] or by its inflammatory [**30**,**31**] and angiogenic component [**32**].

***Fundus autofluorescence***

As mentioned earlier, lipofuscin in the retina shows a characteristic autofluorescent pattern [**22**]. Initially, retinal autofluorescence was assessed by fundus spectrofluorometers, designed to measure excitation and emission spectra from small retinal areas, only 2 degrees in diameter. Fundus fluorescence revealed a maximum emission at 630 nm and peak excitation at 510 nm. It also showed topographic variability across the retinal fundus, with a foveal minimum, a maximum at 7° to 15° from the fovea and a decrease towards the periphery [**33**]. This is determined by a lower amount of lipofuscin in the foveal RPE cells and absorption of the excitation light by macular pigment [**9**].

Fundus autofluorescence imaging has gone a long way since the use of spectrofluorometers and current imaging methods use confocal scanning laser (cSLO) systems and modified fundus cameras, with ultra-wide-field imaging and different excitation wavelengths to evaluate retinal autofluorescence [**34**,**35**]. Currently commercially available cSLO systems used to obtain fundus autofluorescence images are the HRA Spectralis platform (Heidelberg Engineering, Dossenheim, Germany) and the Nidek F-10 Digital Ophthalmoscope (Nidek Co., Ltd., Gamagori, Aichi, Japan). Spectralis HRA+OCT also allows simultaneous cSLO and optical coherence tomography (OCT) recordings [**9**]. The cSLO system is based on excitation of a blue laser, with a wavelength of 488 nm and recording of emitted light above 500 nm.

***Fundus autofluorescence in ophthalmology***

Fundus autofluorescence allows mapping of in vivo lipofuscin distribution across the retinal fundus. This technique is fast, easily repeatable, and noninvasive. Unlike other imaging techniques (fluorescein angiography or indocyanine green angiography) it does not require the use of a fluorescent dye. It is currently regarded as having an important contribution in multimodal imaging, as a complementary imaging technique to fundus photography, angiography, and OCT in the diagnosis of various retinal disease, in determining disease prognosis and evolution over time and in monitoring disease response to treatment [**36**-**40**].

For example, fundus autofluorescence has been used to monitor the idiopathic macular hole.

In eyes with macular hole, the lack of lutein and zeaxanthin pigments in the fovea results in hyperautofluorescence, although hypoautofluorescence is normally noted at the fovea.

## Conclusions

Despite our knowledge regarding lipofuscin composition, its interaction with light and its association with retinal degenerative disease, many questions still exist. 

We must still determine the full spectrum of fluorescent compounds of lipofuscin. We know that A2E is one of them but we also know that its contribution to lipofuscin fluorescence is small. Further characterization of lipofuscin composition, including spectral analysis, may improve current imaging techniques or enhance development of novel ones.

A similar question is asked when we consider the role of A2E in retinal degenerative disease: what component of lipofuscin is pathogenic? An even more complex question is if lipofuscin is at all a cause of retinal degenerative disease or just its consequence. And if progressive accumulation of lipofuscin undeniably leads to retinal damage, is there a way to prevent its formation? 

We also have very limited information about melanolipofuscin granules, their potential usefulness in detecting retinal disease and/ or understanding the pathophysiology of certain disease.

**Conflict of Interest statement**

Authors state no conflict of interest.

**Acknowledgments**

All the authors had equal contributions. 

**Sources of Funding**

No funding was received.

**Disclosures**

Nil.

**Authors’ contributions**

OM and EFC designed the study and are responsible for the acquisition and interpretation of the data.

SIP and CCC were involved in the design of the study, inspecting carefully, and revised the manuscript.

CP was involved in the conception and drafting of the study and revised the manuscript. 

All authors read and approved the final manuscript.

**Authors’ information**

The author Stella Ioana Popescu (Patoni) is a PhD student in the Ophthalmology Department of “Victor Babeş” University of Medicine and Pharmacy in Timișoara, Romania, but she does not work either in the Ophthalmology Department or the faculty. Her affiliation must be this university to have her article validated for her doctoral thesis. Her affiliation with the Ophthalmology Department is because she is a PhD student. 
